# Association among childhood adversity and susceptibility to interference during varying salience: two studies in healthy males

**DOI:** 10.1038/s41598-024-57025-x

**Published:** 2024-03-25

**Authors:** Greta Amedick, Marina Krylova, Kathrin Mayer, Igor Izyurov, Luisa Herrmann, Louise Martens, Vanessa Kasties, Johanna Heller, Meng Li, Johan van der Meer, Ilona Croy, Veronika Engert, Martin Walter, Lejla Colic

**Affiliations:** 1 Department of Psychiatry and Psychotherapy, University Tübingen, Tübingen, Germany; 2 Department of Psychiatry and Psychotherapy, Jena University Hospital, Philosophenweg 3, 07743 Jena, Germany; 3 Department of Radiology, Institute of Diagnostic and Interventional Radiology, Jena University Hospital, Jena, Germany; 4 Max Planck Institute for Biological Cybernetics, Tübingen, Germany; 5 Institute of Clinical Psychology, Center for Mental Health, Hospital Stuttgart, Stuttgart, Germany; 6 QIMR Berghofer Medical Research Institute, Brisbane, Australia; 7 Amsterdam UMC, Department of Radiology and Nuclear Medicine, Amsterdam, The Netherlands; 8 Institute for Psychology, Friedrich Schiller University, Jena, Germany; 9 German Center for Mental Health, partner site Halle-Jena-Magdeburg, Jena, Germany; 10 Institute of Psychosocial Medicine, Psychotherapy and Psychooncology, Jena University Hospital, Jena, Germany; 11 Leibniz Institute for Neurobiology, Magdeburg, Germany; 12 Center for Behavioral Brain Sciences, Magdeburg, Germany

**Keywords:** Adverse childhood experiences, Attention, Interference, Salience, Valence, Hydrocortisone, Salivary alpha-amylases, Stress and resilience, Attention

## Abstract

Childhood adversity, a prevalent experience, is related to a higher risk for externalizing and internalizing psychopathology. Alterations in the development of cognitive processes, for example in the attention-interference domain may link childhood adversity and psychopathology. Interfering stimuli can vary in their salience, i.e. ability to capture attentional focus, and valence. However, it is not known if interference by salience or valence is associated with self-reported adversity. In two independent study samples of healthy men (Study 1: n = 44; mean age [standard deviation (SD)] = 25.9 [3.4] years; Study 2: n = 37; 43.5 [9.7] years) we used the attention modulation task (AMT) that probed interference by two attention-modulating conditions, salience and valence separately across repeated target stimuli. The AMT measures the effects of visual distractors (pictures) on the performance of auditory discrimination tasks (target stimuli). We hypothesized that participants reporting higher levels of childhood adversity, measured with the childhood trauma questionnaire, would show sustained interference in trials with lower salience. Due to conflicting reports on the valence-modulation, we tested the valence condition in an exploratory manner. Linear mixed models revealed an interaction between reported childhood adversity and the salience condition across tone presentations in both study samples (Sample 1: p = .03; Sample 2: p = .04), while there were no effects for the valence condition across both studies. Our study suggests that higher self-reported childhood adversity is related to faster processing of target cues during high salience, but slower during low salience conditions. These results hint to the mechanisms linking childhood adversity and psychopathological symptoms in the attentional domain.

## Introduction

Childhood adversity is a common experience, affecting a high percentage of individuals across countries; for example in Germany it is estimated that between 10 and 30% of individuals have experienced at least one type of adversity before age 18^1,2^. Childhood adversity is defined as abuse, neglect, dysfunctional home or poverty, and they often co-occur^3^. Childhood adversity is related to a higher risk for externalizing and internalizing psychopathology^4^. There are several proposed pathways linking childhood adversity with an increased risk for psychopathology, and one of them is through alterations in cognitive development^5,6^, more specifically in the attention-interference domain^7^.

Interference can be defined as a process that compromises attention on relevant stimuli and degree of susceptibility to interference changes during development^8^. Acute psychosocial stress was shown to increase interference^9^. Thus, adversity as a form of chronic stress during development may shape susceptibility to interference. Indeed, recent meta-analysis indicated that childhood adversity is related to higher interference^10^, and potential mechanism include impaired early learning of cue relevance that can later lead to attentional deficits and psychopathological symptoms^6^. Studies investigating severe adversity in institutionalized children showed that interference during a flanker task mediated the pathway to impulsivity symptoms^11^. Another study reported low resistance to interference in youth with adversity, which further correlated with emotional and behavioral problems^12^. In participants without diagnosed psychopathology, self-reports of childhood adversity were also related to alterations in interference processing^13^.

Interference is influenced by stimulus characteristics. Stimuli can vary in their salience, and stimuli with high salience can be defined as ones that have outstanding features and capture attention. High salient stimuli are also associated with higher interference^14,15^. Next to salience, stimuli can vary in valence. Results from studies suggest that both positively and negatively valenced stimuli increase interference^16,17^, but a recent review suggested that it may depend on the task context^18^. The commonly used method to operationalize interference is through reaction time (RT) models and their modulation by task-irrelevant stimuli^19^.

**Figure 1:**
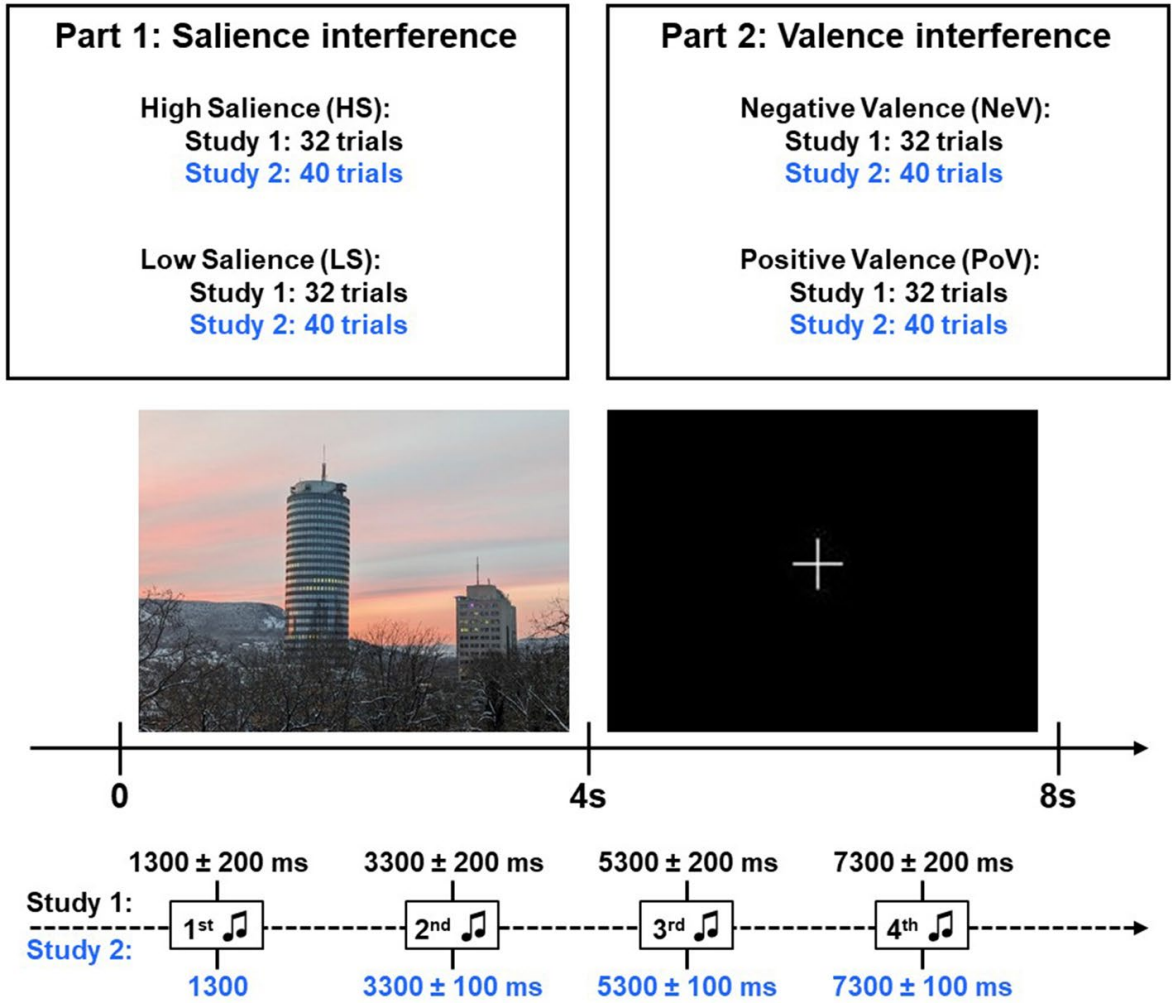
AMT task design. Both studies shared same task design measuring effects of visual distractors (pictures) on the performance of an auditory discrimination task (target stimuli). There were two parts, *salience interference* with two conditions that were measured with high salience and low salience pictures, and *valence interference* with two conditions that were measured with positive and negative valence pictures. During each trial four tones were presented and current analysis focus on the first two tones during picture presentation. Images for this figure were taken by MK while the actual experiment used IAPS pictures. *ms* milliseconds; *s* seconds.

Therefore, to investigate the effects of childhood adversity on interference in the two domains, salience and valence, separately in the same participants, we utilized the attention modulation task (AMT). AMT is a simple auditory discrimination task (target cues), with simultaneous irrelevant pictures (distractor cues; Fig. 1). The task was previously shown to detect interference both in the salience^20,21^ and valence domains^22^. For the salience domain, the high salient pictures were shown to prolong the RT, thereby indicating interference effects, especially during the first tone presentation. In the valence domain, negatively valanced pictures had longer RT compared to the positively valanced ones. In addition, an early unpublished study found comparable effects for visual distractors using AMT and showed that interference was only driven by stimuli salience and is not affected by other factors^23^. We used linear mixed models to test the interaction between conditions (salience or valence), tone presentation and self-reported levels of childhood adversity on RT in two independent study samples of healthy men. We hypothesized that participants reporting higher levels of childhood adversity will show sustained interference, i.e. that in those participants RT will be similarly long across high and low salient conditions and that RT will be longer across tone presentation during high salient condition. Due to conflicting reports on the valence-modulation domain, we tested the interactions in the valence domain in an exploratory manner.

Childhood adversity is also connected to the alterations in components of stress mechanism^24,25^, which may further influence general cognitive functioning and interference. For example, salivary cortisol, the hypothalamic–pituitary–adrenal axis output hormone, was altered in participants with high childhood adversity in both stress reactivity and diurnal regulation, i.e. not only during stress reactivity but also ‘state levels’^26,27^ (although see Ref.^28^). Previous research has additionally indicated that salivary alpha-amylase levels, an enzyme that is used as a proxy measure for sympathetic system^29^, was also altered in diurnal profiles^30^ and stress reactivity^31,32^ in individuals with high adversity. Cortisol and alpha-amylase may affect interference through affecting brain regions involved in early processing (amygdala) and control (prefrontal cortex)^33,34^. Interestingly, levels of salivary cortisol after stress induction were related to interference and faster reaction times^35^. A preliminary study in children also showed that both state salivary cortisol and alpha-amylase modify interference^36^. Therefore, in study 2 we additionally measured ‘state stress hormone levels’ via salivary levels of cortisol and alpha-amylase^37^ and tested their effects on interference in both domains. The analyses were tested in an exploratory manner and considered preliminary as they were measured just in one study sample.

## Results

Table 1 shows two samples’ characteristics.

**Table 1:** Study variables.

	Study 1, mean [SD]	Study 2, mean [SD]
Age	25.9 [3.4]	43.5 [9.8]
CTQ-tot	33.1 [7.1]	33 [4.9]
RT, salience part	1 [0.037]	1 [0.044]
RT, valence part	1 [0.039]	1 [0.050]
Cortisol (median) nmol/L	–	19.6 [12.4]^a^
Alpha-amylase (median) U/mL	–	151 [92.1]^a^

*CTQ*-*tot* total score of the Childhood Trauma Questionnaire (short form); *RT* reaction times (across both time points and conditions); *SD* standard deviation.

^a^one participant missing measurement.

### Effects of childhood adversity on salience interference

**Figure 2:**
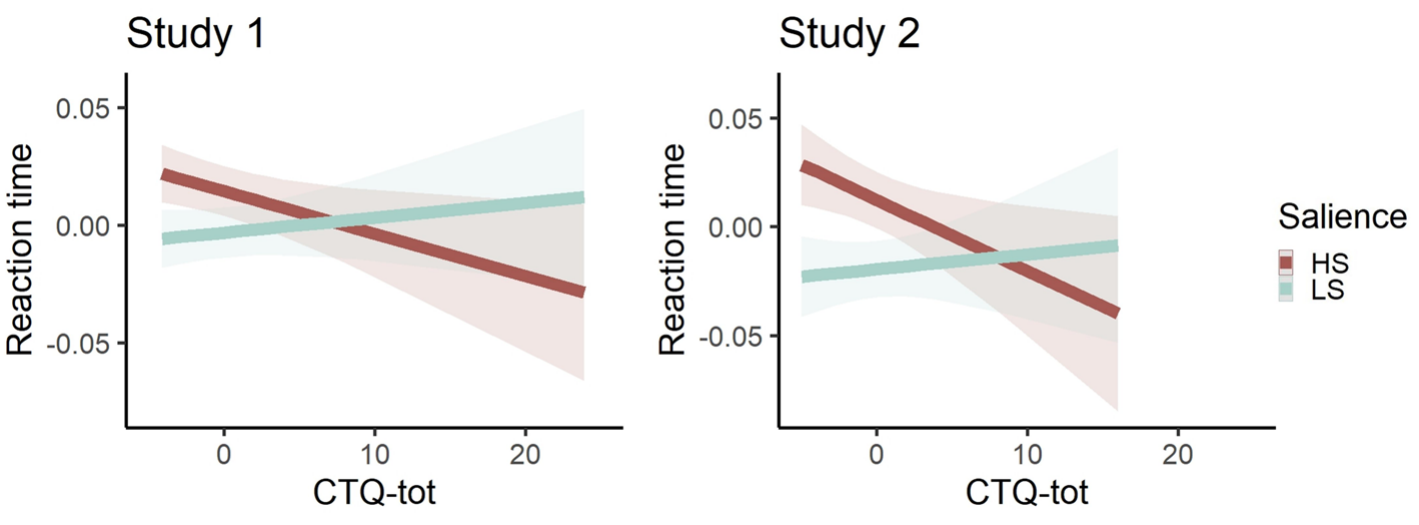
Salience interference by childhood adversity. Across both studies there was a significant interaction between the CTQ-tot and salience condition within the full model. Plots show marginal effects of the interaction term in the full model in each Study. Reaction times and CTQ-tot were grand-mean centered. Follow-up models within each salience condition separately showed a negative association among the childhood adversity and RT in the high salience condition (Study 1: b = − 0.0007, p = 0.19; Study 2: b = − 0.001, p = .20) and positive association among childhood adversity and RT in low salience condition (Study 1: b = 0.0007, p = 0.18; Study 2: b = 0.001, p = 0.24). *CTQ*-*tot* total score of Childhood Trauma Questionnaire; *HS* high salience condition; *LS* low salience condition.

We tested the associations between childhood adversity using the total CTQ-tot score and susceptibility to interference by varying salience, measured with conditions- high and low salience and tone presentation. In both studies in the full model there was a significant interaction between the CTQ-tot and condition (Study 1: b = 0.002, 95% bootstrapped confidence interval (95% CI_bo) [0.0003, 0.005], p = 0.04; Study 2: b = 0.004, 95% CI_bo [0.0007, 0.007], p = 0.03; Fig. 2). Follow-up models within each condition separately, accounting for the main effect of tone presentation, displayed that participants with higher levels of self-reported early life adversity exhibited faster reaction time (RT) during high salient condition (Study 1: b = − 0.0007, 95% CI_bo [− 0.002, 0.0003], p = 0.19; Study 2: b = − 0.0012, 95% CI_bo [− 0.003, 0.0006], p = 0.20), but slower during low salient condition (Study 1: b = 0.0007, 95% CI_bo [− 0.0005, 0.002], p = 0.18; Study 2: b = 0.001, 95% CI_bo [− 0.0008, 0.003], p = 0.24).

Moreover, there was an interaction between the CTQ-tot and tone order (Study 1: b = 0.002, 95% CI_bo [0.0003, 0.004], p = 0.03; Study 2: b = 0.004, 95% CI_bo [− 0.0002, 0.008], p = 0.06), where men with higher levels of self-reported early life adversity showed faster RT during first, and slower during second tone presentation. Full model statistics are presented in Tables S2 and S3. Results were similar when the outliers were excluded (Tables S4 and S5).

### Effects of childhood adversity on valence interference

We tested the associations between childhood adversity using total CTQ-tot score and susceptibility to interference by varying valence, measured with conditions- negative and positive valence and tone presentation. There were no significant interactions except a trend-significant interaction in Study 1 between condition and CTQ-tot (b = 0.002, 95% CI_bo [− 0.0003, 0.005], p = 0.06), where higher CTQ-tot was connected to faster RT during negative valence block. Results for full models and without outliers are shown in Tables S6–S9.

### Effects of salivary ‘state’ cortisol and alpha-amylase on salience and valence interference

In Study 2, we also tested the effects of two ‘state levels of stress hormones’ on the interference. There was a significant interaction among ‘state’ salivary cortisol (b = − 0.002, 95% CI_bo [− 3.31e–03, − 6.32e–05], t = − 2.12, p = 0.037). Post-hoc tests indicated that the effect was driven by the negative association among ‘state’ salivary cortisol and RT during high salience tone 1 (b = − 0.0009, 95% CI_bo [− 0.002, 0.0001], t = − 1.75, p = 0.089; other post-hoc tests p > 0.35). There were no significant interaction effects for ‘state’ salivary cortisol during the *valence interference part* or ‘state’ salivary alpha-amylase for both parts. Results are shown in Tables S10–S13.

## Discussion

Childhood adversity affects interference, yet it is unknown if the effects are based on the salience of valence of the interfering cues. By dissociating these effects, we may have a better understanding of the affected systems by childhood adversity and whether the previously described effects of negative valence (e.g. angry faces) may have been better attributed to high salience. In the current study, we investigated interactions between self-reported childhood adversity and interference by varying the salience and valence of interfering cues. We detected significant effects of salience but not valence across two different study samples of healthy men. In more detail, we observed that higher adversity was associated with faster reaction time (RT) during highly salient cues, but slower RT during low salient cues. There was also an interaction between adversity and tone presentation order, where higher adversity was related to faster RT during first, and slower RT during second tone presentation (across both high and low salience).

### Salience interference

The current results indicate that adversity primarily interacts with the salience of distractor cues. Partially supporting our hypothesis, the interaction showed that during the presentation of low salience pictures, participants with higher self-reported adversity showed longer RT. We therefore speculate that the participants with adversity may have problems with salience attribution. Supporting our speculation, a task explicitly testing the salience domain showed that adversity is connected to reduced adaptive salience processing during learning of stimulus-reward association^38^. Other studies indirectly support our results. For instance, healthy participants with mild childhood adversity (neglect) showed reduced interference monitoring^39^ that may indicate lower discrimination between congruent and incongruent (more salient) trials. In an oddball experiment, the differences between young adults with high and low adversity were present in the percentage of error rates, especially during the predictable blocks^40^, which shows parallels with the low salience condition realized here. Another recent study reported that brain response during novel, salient stimuli in the oddball paradigm mediated the relationship to externalizing symptoms in youth^41^. We also detected an effect of adversity and time on the RT, where during the first tone presentation participants with higher adversity had faster, while during the second tone presentation they exhibited slower reactions. We explain this observation with an attentional shift towards distractor images during the first compared to the second tone, which may have been adaptive for participants with adversity. Longer RT for the second tone may be attributed to a state of general hyperarousal associated to aberrant salience processing^42^. Another explanation may be that there is a broadening of attention/monitoring of distractor stimuli during the second tone which is indicated via longer RT^43,44^.

### Valence interference

We did not observe significant effects across both study samples for valence condition (only trend significant interaction in study sample 1). The lack of consistent association reflects the current literature on adversity-related susceptibility to valence, which have been mixed. Studies using faces as a source of valance information, found higher engagement with negative valence faces in children and adults with high adversity for both target and distractor cues, reflecting general attendance to negative valence^45,46^. In a study that probed valence interference using a flanker task, participants with higher adversity had more similar reaction time (RT) across congruent and incongruent trials for negative valence compared to positive and neutral^47^, indicating emotional ‘priming’ by negative valence. In contrast, a recent study found no interaction between adversity and levels of valence interference in the affective Stroop task in youth^48^. Some authors posit that the effects of childhood adversity on interference during behavioral tasks may be explained through increased vigilance for threat cues^49^. Evidence for increased focus on threat stimuli (mostly indexed by angry faces) comes from studies which reported that children and adults with adversity are more attentive to angry faces^50,51^, although there are also negative findings^52^. Because the negatively and positively valanced pictures were matched for salience levels, it may be that the previously observed effects were an underlying effect of salience information (such as for angry faces).

### ‘State’ salivary hormone levels

We observed a negative association among ‘state’ salivary cortisol levels and RT during high salience and first tone presentation. The result indicates that higher cortisol reduces salience interference. The literature on the association of cortisol and interference or other cognitive processes such as inhibition is mixed and predominantly related to either cortisol reactivity after a stress induction or pharmacological manipulation. For example, a meta-analysis that looked at moderating effects of cortisol administration on different type of cognitive processes including interference did found any overall effects of cortisol administration^53,54^, although authors suggested that there may be differences in effects of cortisol across time and that fast effects of cortisol administration (i.e. circulating cortisol) may increase cognitive inhibition^54^. Moreover, studies that measured pre- and post-cortisol levels after stress induction also reported conflicting results; some studies reported faster RT with higher cortisol^35^ while others reported no association among salivary cortisol or alpha-amylase levels and interference measures (accuracy or RT)^55–57^. In our study we measured non-manipulated ‘state’ level of salivary cortisol. We speculate that normally fluctuating endogenous levels of cortisol may therefore “boost” fast reactivity for salient stimuli. However, our speculation needs further testing in naturalistic designs with multiple concomitant assessment of salivary hormone levels and cognitive interference. In contrast, we did not observe interactions of ‘state levels’ of salivary alpha-amylase with salience or valence interference. The discrepancy from studies that report association may stem from the difference in study populations (i.e. Ref.^36^ investigated children).

### Limitations and future direction

The current studies included only healthy men. Population surveys indicate some differences in prevalence and types of childhood adversities between the genders^1,58^, so studies including a gender-diverse population confirming our results are needed. Moreover, we measured childhood adversities in the context of abuse and neglect and did not account for other aspects such as socio-economic status during childhood or family dysfunction^59^. A more holistic approach to determining childhood adversity, including timing and severity is suggested to discerning effects of adversity type on the interference processes. Testing the AMT task in participants that exhibit moderate to severe psychopathological symptoms may also help in identifying mediating links between childhood adversity, salience interference and presence of psychopathology. The current task aimed to discern effects specific to domain of salience or valence while controlling for the effects of the other domain (Fig. S1). Nevertheless, we acknowledge that the processing of the two domains remains interconnected^60^. Some studies in humans^61^ and animals^62^ suggest that during behaviors (e.g. reward or fear) both domains are processed simultaneously in amygdala. Thus, including complementary measurements using skin conductance, eye tracking and concomitant subjective ratings may further dissociate effects childhood adversity on processing of salience and valence and the following interference effects. In particular, it may help discern effects of childhood adversity on (non)specific to *salience-* or *valence-interference* processing depth and arousal^63,64^. Moreover, the result concerning the effect of ‘state’ cortisol should be considered preliminary as we measured salivary hormones only in Study 2 and need further replication. In sum, in two separate study samples of healthy men we tested the effects of self-reported childhood adversity on susceptibility to salience and valence cues in interference. Childhood adversity interacted with the effect of condition during salience but not valence suggesting that higher self-reported childhood adversity may be related to faster processing during high salience and slower processing of target cues during low salience condition. Our results suggest potential mechanisms that links childhood adversity and symptoms in the attentional domain reported by epidemiological studies.

## Methods

### Participants

Participants were recruited for two independent studies. Study 1 was designed to investigate the effects of childhood adversity on cognitive processing and stress responsivity and was conducted at the University Hospital Tuebingen. Forty-six healthy male participants were included in Study 1. Two participants did not complete the attention modulation task (AMT) task so forty-four were included in the current data analysis (age range 20–32 years, mean age [standard deviation (SD)] = 25.9 [3.4] years).

Study 2 was a cross-over clinical trial (NCT02602275; date of registration: 11/11/2015) in which participants took either placebo or Neurexan in a counterbalanced order. Neurexan (Nx4) is a medicinal product, consisting of three herbal extracts (Avena sativa, Coffea arabica, Passiflora incarnata) and one mineral salt (Zincum isovalericum). According to the trial protocol participants completed a set of procedures and tasks and the results of the outcomes (treatment effects) of the trial are reported elsewhere (see Refs.^65–68^. The following research question was not registered as part of the outcomes of the trial and was considered as a complementary replication sample to Study 1. Thirty-nine healthy male participants completed the study. Two participants were excluded as outliers in the processing step (please see below). Therefore, thirty-seven participants were included in the current data analysis (age range 31–59 years, mean age [standard deviation (SD)] = 43.5 [9.7] years). Due to possible repetition effects when participants complete the AMT task for a second time, we restricted our analyses to the first-time completion, and pooled the data across conditions to measure the effect orthogonal to treatment. Study 2 was conducted at the Otto von Guericke University Magdeburg.

Participants in both studies were assessed with the German Version 5.0.0 of the Mini International Neuropsychiatric Interview (M.I.N.I.)^69,70^, to ensure the absence of current psychiatric illness according to the Diagnostic and Statistical Manual of Mental Disorders 4th edition (DSM-IV)^71^. Participants were without medication, determined by the medical history interview. Other exclusion criteria were neurological illness, other major medical illnesses (e.g. diabetes) and magnetic resonance contraindications. All participants were right-handed, measured with the short form of the Edinburgh Handedness Inventory^72^. Both studies were conducted in accordance with the Declaration of Helsinki and were approved by the Institutional Review Board of the University of Tuebingen (Study 1) or the University of Magdeburg (Study 2). All participants gave written informed consent and were reimbursed for their participation.

### Childhood adversity

In both studies childhood adversities were assessed using the German version of the Short Form of the childhood trauma questionnaire (CTQ-SF; Refs.^73,74^). The CTQ-SF is a self-reported retrospective questionnaire that assesses five types of adverse childhood experiences: physical, emotional, and sexual abuse and physical and emotional neglect. Each scale consists of five items rated with a five-point Likert scale ranging from 1 (never true) to 5 (very often true), and severity scores range from 5 to 25. Good reliability, validity, and item consistency have been demonstrated for the German version of the CTQ^75^. Total scores (CTQ-tot) were used in the current analyses (Study 1: range 25–61, mean [SD] = 33.1 [7.1]; Study 2: range 25–49, mean [SD] = 33.0 [4.9]).

### Cortisol and alpha-amylase quantification

In study 2 saliva cortisol and alpha-amylase were measured at eight time points during the measurement day in the laboratory to assessing the ‘state levels of stress hormones’ and stress hormones reactivity not related to this study (see Ref.^68^). For the analysis here, two measurements, before and after the AMT task were considered, and a median value was calculated. We used a median measure to capture the overall ‘state level’ of salivary cortisol and alpha-amylase across the AMT. The measurements took place in the afternoon (between 15.00 and 18.50 h) and the time between the two measurements was mean [SD] = 59 [21] min. Participants followed the instructions for the sampling that included controlled food intake. Saliva samples were collected with Salivettes^®^ (Sarstedt, Nuembrecht, Germany). Salivary cortisol levels were analyzed using a commercial enzyme-linked immunosorbent assay (Cortisol ELISA, IBL International, Hamburg, Germany) according to the manufacturer’s instructions. Intra- and inter-assay variances were 4.8% and 5.9%, respectively. Median cortisol was 19.6 [12.4] nmol/L (range 3.92–51.4). Salivary alpha-amylase activity was determined using a commercially available enzymatic assay according to the manufacturer’s instructions. Briefly, diluted saliva (1:200) was mixed with a prewarmed (37 °C) solution of 2-chloro-p-nitrophenol linked to maltotriose. The enzymatic conversion of this substrate by alpha-amylase yields 2-chloro-p-nitrophenol, which can be spectrophotometrically measured at 405 nm. The increase in absorbance at 405 nm over a period of two min is directly proportional to the amount of alpha-amylase activity present in the sample. Intra- and inter-assay variances were 2.4% and 3.5%, respectively. The quantification was not possible for one participant due to lack of saliva. The unit of alpha-amylase was (µmol/s)/L and the values were then converted to U/mL with a conversion factor of 0.06. Median alpha-amylase was 151 [92.1] U/mL (range 17.8–386).

### Interference assessment with the AMT task

The attention modulation task (AMT) measures the effects of visual distractors (pictures) on the performance of an auditory discrimination task (target stimuli). The goal for the participants was to discriminate tones while passively looking at the distractor pictures. Participants heard ascending (600–720 Hz) or descending (600–500 Hz) tones via headphones and were instructed to press the left mouse button (right index finger) for ascending and right mouse button (right middle finger) for descending tones as fast as possible. The tone modulations were created using MATLAB v.7.1 (R2005b). Two studies differed slightly in their design and will be marked as Study 1/Study 2. One trial lasted 8066/ 8000 ms and included a distractor picture (4033/4000 ms) followed by a white fixation cross on a black screen (4000 ms). During both picture and fixation cross two tones were presented (one trial, total 4 tones). First tone was presented 1300 ± 200/1300 ms after the trial onset while the next tones were presented with variable intervals of 2000 ± 200/ 2000 ± 100 ms to avoid habituation. Each tone lasted for 300 ms. Tone types and inter-tone intervals were randomized and balanced across pictures and tone onsets. The pictures were standardized, colored and taken from the International Affective Picture System (IAPS; Ref.^76^). In total there were one hundred twenty-eight/one hundred and sixty trials in two parts with two conditions in each part. In the first part of the task, the *salience interference*, two conditions were measured via thirty-two/ forty trials with high salience pictures (HS; sixteen/ twenty pictures with erotic content) and via thirty-two/ forty trials with low salience pictures (LS). In the second part of the task, *valence interference,* two conditions were measured via thirty-two/ forty trials with negative valence pictures (NeV) and thirty-two/ forty trials with positive valence pictures (PoV). HS and LS were matched for valence (all pictures were with positive valence) and NeV and PoV were matched for salience. Salience and valence ratings were conducted in Study 1 and are added in Supplementary (Table S1 and Fig. S1). The trials within each part and condition were randomized and the same across all participants. Accuracy and reaction times (RT) were recorded. The task was presented via Presentation Software (Neurobehavioral Systems, Inc., San Francisco, CA). Participants were seated in a quiet room with turned off lights, to rule out environmental influences. Additionally, in Study 2 electroencephalography was recorded during the task. The AMT task has been validated and previously published in other studies^20,21,23^ and in an EEG-AMT analysis of Study 2 effects of NX4 have been reported^22^.

### Data analysis

#### Reaction time processing

In both studies the same processing and statistical analysis were used. Following previous research, distraction was measured with RT in both parts regardless of tone type (ascending or descending). RT were processed as follows. Trials with incorrect discrimination, anticipatory RT < 100 ms and prolonged (lack of concentration) RT > 1800 ms (i.e. RT as long as the inter-stimulus interval) were excluded from processing. Then, in each part separately median RT were calculated per condition and tone order (*salience interference part*: high salient-tone order 1; high salient-tone order 2; low salient-tone order 1; low salient-tone order 2; *valence interference part:* negative valence-tone order 1; negative valence-tone order 2; positive valence-tone order 1; positive valence-tone order 2). To controlling task-unspecific intraindividual variability^77^ median RT were normalized to individual (participant’s) mean median RT across the *salience* or *valence interference* part separately. In Study 2 two participants did not complete the AMT task.

### Statistical analysis

To examine whether childhood adversity impacted RT during *salience* or *valence interference* we used linear mixed models for each part separately. RT (grand-mean centered) were analyzed as a function of childhood adversity (CTQ-tot, grand-mean centered), tone order (tone 1 vs tone 2), and condition (HS vs LS, or NeV vs PoV). Participants were considered as a random effect and random intercept and random slopes of both condition (salience or valence) and tone order were estimated. We investigated main and interaction effects of childhood adversity with tone order, condition or tone order by condition. Significant interaction effects were followed by post-hoc models within each condition separately (high salient and low salient; negative valence and positive valence), in which main effect of childhood adversity was checked accounting for the main effect of tone order. For study 2, median salivary cortisol and alpha-amylase values, indicating ‘state levels of stress hormones’, were tested in the same manner. Mixed models were estimated using Satterthwaite approximate degrees of freedom and estimates were obtained by restricted maximum likelihood estimation. Models had an unstructured variance–covariance matrix. Normality of the variance was checked via residual plots. The alpha value was set to 0.05. Linear mixed models (with diagnostics and plotting) were run with ‘lme4’, ‘lmerTest’, ‘sjPlot’, ‘multilevelTools’ and ‘car’, while graphs with ‘ggplot2’ package in R (V4.1.3). To test if the observed effects may have been driven by outliers, we ran linear mixed models without outliers for RT, CTQ-tot, and hormones detected via the Rosner test (‘EnvStats’ package).

### Supplementary Information

Supplementary Information.

## Data Availability

The datasets generated and/or analysed during the current study are not publicly available due to data protection laws and consent that participants gave but processed data are available from the corresponding author on reasonable request.
