# Supplementary material for: Association among childhood adversity and susceptibility to interference during varying salience: two studies in healthy males
Source: Sci Rep. 2024 Mar 25;14:7050. doi: 10.1038/s41598-024-57025-x (PMC10963761; doi:10.1038/s41598-024-57025-x)
Supplement: Supplementary file 1 — Supplementary Information. [file 41598_2024_57025_MOESM1_ESM.docx]

# Supplementary Results

## ***Interference effects in salience and valence parts***

In both study samples, as a proof of principle, we tested the effects of interference of picture conditions on the RT. We applied linear mixed models for each part (salience or valence) separately. RT (mean-centered) were analyzed as a function of tone order (tone 1 vs tone 2), and condition (HS vs LS / NeV vs PoV). Participants were considered as a random effect and random intercept and random slopes accounting for condition and tone order were estimated. We investigated main and interaction effects of tone order, condition or tone order by condition. Mixed models were run as described in the methods.

For the *salience interference part* in both studies there were a main effect of salience where RT were longer during high salient pictures i.e., suggesting interference effects (Study 1: Condition [LS], b= -0.018, CI_bo [95%]= [-33.3e-3, -0.53e-3], t= -2.10, p= 0.039; Study 2: Condition [LS], b= -0.032, CI_bo [95%]= [-0.049, -0.016], t= -3.74, p< .001). There were no interaction effect in Study 1 (Tone order [Tone 2] by Condition [LS], b= 0.002, CI_bo [95%]= [-0.015, 0.019], t= 0.28, p= 0.78). In contrast, in Study 2 there were an interaction effect (Tone order [Tone 2] by Condition [LS], b= 0.067, CI_bo [95%]= [0.047, 0.087], t= 6.65, p< .001).

In study 2, post-hoc tests indicated that in both conditions there were differences among tone presentations, where in the high salient condition the RT were longer during the first tone presentation (Tone order [Tone 2], b= -0.026, CI_bo [95%]= [-0.042, -0.007] t= -2.89, p= .005) while in the low salient condition the RT were longer during the second tone presentation (Tone order [Tone 2], b= 0.041, CI_bo [95%]= [0.024, 0.059], t= 4.17, p< .001).

For the *valence interference part,* the results were not consistent. In Study 1 there were no significant main effects of valence (Condition [POS], b= -0.005, CI_bo [95%]= [-0.025, 0.012], t= -0.61, p= .54) or significant interaction effects (Tone order [Tone 2] by Condition [POS], b= 0.004, CI_bo [95%]= [-0.010, 0.017], t= 0.54, p= .59). In contrast, in Study 2 there were main effect of valence indicating longer RT and interference by negative pictures (Condition [POS], b= -0.033, CI_bo [95%]= [-0.055, -0.008], t= -2.79, p= .007). In Study 2 there were also an interaction effect (Tone order [Tone 2] by Condition [POS], b= 0.070, CI_bo [95%]= [0.051, 0.091], t= 6.74, p< .001) where the post-hoc indicated that during the negative picture condition RT were longer during the first tone (Tone order [Tone 2], b= -0.064, CI_bo [95%]= [-0.089, -0.039], t= -5.43, p< .001) which was not present during the positive condition (Tone order [Tone 2], b= 0.006, CI_bo [95%]= [-0.008, 0.019], t= 0.77, p= .45).

## ***Salience and valence ratings in Study 1***

In the salience interference part, high and low salience conditions were matched for valence ratings, and both had positive valence. In the valence interference condition, positive and negative conditions were matched for salience ratings. In Study 1, participants rated pictures on two dimensions. Questions were “The picture is salient” (“Das gezeigte Bild ist salient”), with a scale “not at all [10] - extremely [90]” and “What kind of feeling did you experience [negative-neutral-positive]” (“Welche ART von Gefühl hast du erlebt [Negativ-Neutral-Positiv]”), with a scale Negativ [10] - Neutral [50] - Positiv [90].

Due to technical mistake, all participants did not rate one high salience picture, thus in total 127 pictures were rated. One participant rated only 60 pictures. For each picture ratings were averaged across participants. Table S1 shows mean ratings for each condition, and Figure S1 shows dispersion of mean ratings for each picture across the two dimensions (salience and valence).

Table S1. Mean picture ratings across the four conditions.

|  | Salience (mean ± SD) | Valence (mean ± SD) |
| --- | --- | --- |
| *Salience interference* |  |  |
| High salience | 62.7 ± 8.6 | 61.5 ± 8.6 |
| Low salience | 39.4 ± 7.9 | 53.6 ± 6.0 |
| *Valence interference* |  |  |
| Negative valence | 59.5 ± 9.6 | 32.3 ± 6.5 |
| Positive valence | 50.3 ± 5.7 | 62.1 ± 5.2 |

Abbreviations: SD= standard deviation

Table S2. Linear mixed model estimates for the CTQ-tot, Tone and Condition on the RT for *salience interference*, Study 1.

| Fixed effects |  |  |  |  |
| --- | --- | --- | --- | --- |
|  | b | CI_bo [95%] | t | p |
| (intercept) | 1.4e-02 | [0.004, 0.02] | 2.68 | 0.008 |
| Tone order [Tone 2] | -1.3e-02 | [-0.03, 0.001] | -1.81 | 0.072 |
| Condition [LS] | -1.7e-02 | [-0.03, -0.002] | -2.13 | 0.037 |
| CTQ-tot | -1.8e-03 | [-0.003, -0.0004] | -2.35 | 0.020 |
| Tone order [Tone 2] by Condition [LS] | 2.4e-03 | [-0.01, 0.02] | 0.28 | 0.78 |
| **Tone order [Tone 2] by CTQ-tot** | **2.1e-03** | [0.0003, 0.004] | **2.19** | **0.031** |
| **Condition [LS] by CTQ-tot** | **2.4e-03** | [0.0003, 0.005] | **2.07** | **0.042** |
| Tone order [Tone 2] by Condition [LS] by CTQ-tot | -1.9e-03 | [-0.004, 0.0005] | -1.62 | 0.11 |
| Random effects |  |  |  |  |
| Groups | Variance | Standard deviation |  |  |
| Participant (intercept) | 0.0001 | 0.011 |  |  |
| Participant (time) | 0.0005 | 0.023 |  |  |
| Participant (intercept) | 0.0003 | 0.019 |  |  |
| Participant (condition) | 0.001 | 0.038 |  |  |
| Residual | 0.0008 | 0.028 |  |  |
| Observations | 176 |  |  |  |
| Participants | 44 |  |  |  |

Table S3. Linear mixed model estimates for the CTQ-tot, Tone and Condition on the RT for *salience interference*, Study 2.

| Fixed effects |  |  |  |  |
| --- | --- | --- | --- | --- |
|  | b | CI_bo [95%] | t | p |
| (intercept) | 0.01 | [0.0004, 0.02] | 1.87 | 0.065 |
| Tone order [Tone 2] | -0.03 | [-0.05, -0.006] | -2.58 | 0.012 |
| Condition [LS] | -0.03 | [-0.05, -0.02] | -3.80 | 0.0003 |
| CTQ-tot | -0.003 | [-0.006, -0.0007] | -2.38 | 0.020 |
| Tone order [Tone 2] by Condition [LS] | 0.07 | [0.05, 0.09] | 6.66 | <0.001 |
| **Tone order [Tone 2] by CTQ-tot** | **0.004** | **[-0.0002, 0.008]** | **1.95** | **0.056** |
| **Condition [LS] by CTQ-tot** | **0.004** | **[0.0007, 0.007]** | **2.26** | **0.027** |
| Tone order [Tone 2] by Condition [LS] by CTQ-tot | -0.003 | [-0.007, 0.001] | -1.47 | 0.14 |
| Random effects |  |  |  |  |
| Groups | Variance | Standard deviation |  |  |
| Participant (intercept) | 0.0005 | 0.022 |  |  |
| Participant (time) | 0.002 | 0.044 |  |  |
| Participant (intercept) | 0.0002 | 0.013 |  |  |
| Participant (condition) | 0.0007 | 0.027 |  |  |
| Residual | 0.0009 | 0.0304 |  |  |
| Observations | 148 |  |  |  |
| Participants | 37 |  |  |  |

Table S4. Linear mixed model estimates for the CTQ-tot, Tone and Condition on the RT for *salience interference*, outliers excluded, Study 1.

| Fixed effects |  |  |  |  |
| --- | --- | --- | --- | --- |
|  | b | CI_bo [95%] | t | p |
| (intercept) | 8.73 | [-15.15, 31.56] | 0.77 | 0.45 |
| Tone order [Tone 2] | -5.82 | [-13.93, 1.90] | -1.46 | 0.15 |
| Condition [LS] | -8.23 | [-16.17, 0.68] | -1.89 | 0.062 |
| CTQ-tot | 1.55 | [-8.68, 0.88] | 0.35 | 0.72 |
| Tone order [Tone 2] by Condition [LS] | -0.16 | [-11.96, 11.58] | -0.03 | 0.98 |
| **Tone order [Tone 2] by CTQ-tot** | **1.62** | **[-1.34, 4.77]** | **1.06** | **0.29** |
| **Condition [LS] by CTQ-tot** | **3.54** | [0.47, 6.96] | **2.12** | **0.037** |
| Tone order [Tone 2] by Condition [LS] by CTQ-tot | -4.17 | [-8.75, 0.57] | -1.94 | 0.057 |
| Random effects |  |  |  |  |
| Groups | Variance | Standard deviation |  |  |
| Participant (intercept) | 340.84 | 18.46 |  |  |
| Participant (time) | 113.07 | 10.63 |  |  |
| Participant (intercept) | 4166.28 | 64.55 |  |  |
| Participant (condition) | 0.83 | 0.91 |  |  |
| Residual | 287.64 | 16.96 |  |  |
| Observations | 147 |  |  |  |
| Participants | 37 |  |  |  |

Table S5. Linear mixed model estimates for the CTQ-tot, Tone and Condition on the RT for *salience interference*, outliers excluded, Study 2.

| Fixed effects |  |  |  |  |
| --- | --- | --- | --- | --- |
|  | b | CI_bo [95%] | t | p |
| (intercept) | 9.01 | [-18.56, 39.01] | 0.63 | 0.53 |
| Tone order [Tone 2] | -13.68 | [-26.02, -3.18] | -2.22 | 0.029 |
| Condition [LS] | -18.40 | [-29.21, -7.55] | -3.29 | 0.002 |
| CTQ-tot | 2.35 | [-4.28, 9.07] | 0.67 | 0.51 |
| Tone order [Tone 2] by Condition [LS] | 36.75 | [21.24, 53.11] | 4.68 | <0.001 |
| **Tone order [Tone 2] by CTQ-tot** | **3.45** | [0.19, 6.09] | **2.26** | **0.026** |
| **Condition [LS] by CTQ-tot** | **2.23** | **[-0.42, 4.84]** | **1.61** | **0.11** |
| Tone order [Tone 2] by Condition [LS] by CTQ-tot | -2.30 | [-5.77, 1.42] | -1.18 | 0.24 |
| Random effects |  |  |  |  |
| Groups | Variance | Standard deviation |  |  |
| Participant (intercept) | 6667.52 | 81.65 |  |  |
| Participant (time) | 15.15 | 3.89 |  |  |
| Participant (intercept) | 26.71 | 5.17 |  |  |
| Participant (condition) | 253.66 | 15.93 |  |  |
| Residual | 554.76 | 23.55 |  |  |
| Observations | 144 |  |  |  |
| Participants | 36 |  |  |  |

Table S6. Linear mixed model estimates for the CTQ-tot, Tone and Condition on the RT for *valence interference*, Study 1.

| Fixed effects |  |  |  |  |
| --- | --- | --- | --- | --- |
|  | b | CI_bo [95%] | t | p |
| (intercept) | 2.6e-03 | [-0.009, 0.01] | 0.43 | 0.66 |
| Tone order [Tone 2] | -1.7e-03 | [-0.02, 0.01] | -0.21 | 0.83 |
| Condition [POS] | -5.5e-03 | [-0.02, 0.01] | -0.62 | 0.54 |
| CTQ-tot | -8.9e-04 | [-0.002, 0.0008] | -1.06 | 0.29 |
| Tone order [Tone 2] by Condition [POS] | 4.0e-03 | [-0.01, 0.02] | 0.54 | 0.59 |
| Tone order [Tone 2] by CTQ-tot | -1.9e-05 | [-0.002, 0.002] | -0.02 | 0.98 |
| **Condition [POS] by CTQ-tot** | **2.4e-03** | [-0.0003, 0.005] | **1.89** | **0.064** |
| Tone order [Tone 2] by Condition [POS] by CTQ-tot | -1.2e-03 | [-0.003, 0.0008] | -1.11 | 0.27 |
| Random effects |  |  |  |  |
| Groups | Variance | Standard deviation |  |  |
| Participant (intercept) | 0.0004 | 0.019 |  |  |
| Participant (time) | 0.001 | 0.038 |  |  |
| Participant (intercept) | 0.0006 | 0.024 |  |  |
| Participant (condition) | 0.002 | 0.048 |  |  |
| Residual | 0.0006 | 0.025 |  |  |
| Observations | 176 |  |  |  |
| Participants | 44 |  |  |  |

Table S7. Linear mixed model estimates for the CTQ-tot, Tone and Condition on the RT for *valence interference*, Study 2.

| Fixed effects |  |  |  |  |
| --- | --- | --- | --- | --- |
|  | b | CI_bo [95%] | t | p |
| (intercept) | 0.03 | [0.01,0.04] | 4.12 | <0.001 |
| Tone order [Tone 2] | -0.06 | [-0.08, -0.04] | -6.97 | <0.001 |
| Condition [POS] | -0.03 | [-0.05, -0.008] | -2.76 | 0.008 |
| CTQ-tot | -0.0003 | [-0.003, 0.003] | -0.18 | 0.86 |
| Tone order [Tone 2] by Condition [POS] | 0.07 | [0.05, 0.09] | 6.68 | <0.001 |
| Tone order [Tone 2] by CTQ-tot | 0.0002 | [-0.003, 0.004] | 0.12 | 0.91 |
| Condition [POS] by CTQ-tot | 0.001 | [-0.004, 0.006] | 0.50 | 0.62 |
| Tone order [Tone 2] by Condition [POS] by CTQ-tot | -0.002 | [-0.006, 0.002] | -0.85 | 0.40 |
| Random effects |  |  |  |  |
| Groups | Variance | Standard deviation |  |  |
| Participant (intercept) | 0.0003 | 0.017 |  |  |
| Participant (time) | 0.001 | 0.033 |  |  |
| Participant (intercept) | 0.0008 | 0.028 |  |  |
| Participant (condition) | 0.003 | 0.057 |  |  |
| Residual | 0.001 | 0.032 |  |  |
| Observations | 148 |  |  |  |
| Participants | 37 |  |  |  |

Table S8. Linear mixed model estimates for the CTQ-tot, Tone and Condition on the RT for *valence interference*, outliers excluded, Study 1.

| Fixed effects |  |  |  |  |
| --- | --- | --- | --- | --- |
|  | b | CI_bo [95%] | t | p |
| (intercept) | 0.005 | [-0.008, 0.02] | 0.75 | 0.46 |
| Tone order [Tone 2] | -0.004 | [-0.02, 0.01] | -0.49 | 0.62 |
| Condition [POS] | -0.009 | [-0.03, 0.009] | -0.98 | 0.33 |
| CTQ-tot | -0.002 | [-0.007, 0.003] | -0.89 | 0.37 |
| Tone order [Tone 2] by Condition [POS] | 0.008 | [-0.007, 0.02] | 1.03 | 0.31 |
| Tone order [Tone 2] by CTQ-tot | 0.003 | [-0.002, 0.01] | 1.17 | 0.25 |
| Condition [POS] by CTQ-tot | 0.004 | [-0.003, 0.01] | 1.22 | 0.23 |
| Tone order [Tone 2] by Condition [POS] by CTQ-tot | -0.007 | [-0.01, -0.001] | -2.56 | 0.012 |
| Random effects |  |  |  |  |
| Groups | Variance | Standard deviation |  |  |
| Participant (intercept) | 0.0003 | 0.018 |  |  |
| Participant (time) | 0.001 | 0.035 |  |  |
| Participant (intercept) | 0.0005 | 0.023 |  |  |
| Participant (condition) | 0.002 | 0.047 |  |  |
| Residual | 0.0005 | 0.023 |  |  |
| Observations | 148 |  |  |  |
| Participants | 37 |  |  |  |

Table S9. Linear mixed model estimates for the CTQ-tot, Tone and Condition on the RT for *valence interference*, outliers excluded, Study 2.

| Fixed effects |  |  |  |  |
| --- | --- | --- | --- | --- |
|  | b | CI_bo [95%] | t | p |
| (intercept) | 11.81 | [-20.5, 40.8] | 0.77 | 0.44 |
| Tone order [Tone 2] | -26.90 | [-38.0, -16.6] | -5.26 | 1.95e-06 |
| Condition [POS] | -11.73 | [-22.8, 0.4] | -1.93 | 0.058 |
| CTQ-tot | 3.266 | [-3.5, 10.9] | 0.86 | 0.39 |
| Tone order [Tone 2] by Condition [POS] | 29.07 | [16.7, 40.3] | 4.91 | 2.22e-05 * |
| Tone order [Tone 2] by CTQ-tot | 0.64 | [-1.8, 3.1] | 0.51 | 0.61 |
| Condition [POS] by CTQ-tot | 0.97 | [-2.3, 3.8] | 0.65 | 0.51 |
| Tone order [Tone 2] by Condition [POS] by CTQ-tot | -2.09 | [-5.1, 0.6] | -1.43 | 0.16 |
| Random effects |  |  |  |  |
| Groups | Variance | Standard deviation |  |  |
| Participant (intercept) | 5459.2 | 73.89 |  |  |
| Participant (time) | 673.5 | 25.95 |  |  |
| Participant (intercept) | 2665.6 | 51.63 |  |  |
| Participant (condition) | 290.2 | 17.04 |  |  |
| Residual | 305.9 | 17.49 |  |  |
| Observations | 142 |  |  |  |
| Participants | 36 |  |  |  |

Table S10. Linear mixed model estimates for the median cortisol, Tone and Condition on the RT for *salience interference*, Study 2.

| Fixed effects |  |  |  |  |
| --- | --- | --- | --- | --- |
|  | b | CI_bo [95%] | t | p |
| (intercept) | 0.01 | [-3.9e-03, 0.02] | 1.67 | 0.099 |
| Tone order [Tone 2] | -0.02 | [-4.5e-02, -0.0005] | -2.34 | 0.023 |
| Condition [LS] | -0.03 | [-4.6e-02, -0.01] | -3.61 | 0.0006 |
| Cortisol | -0.0009 | [-1.9e-03, 0.0001] | -1.64 | 0.10 |
| Tone order [Tone 2] by Condition [LS] | 0.07 | [4.6e-02, 0.09] | 6.55 | 9.2e-09 |
| Tone order [Tone 2] by Cortisol | 0.001 | [-2.2e-04, 0.003] | 1.64 | 0.10 |
| **Condition [LS] by Cortisol** | **0.001** | **[-1.0e-05, 0.003]** | **1.83** | **0.072** |
| **Tone order [Tone 2] by Condition [LS] by Cortisol** | **-0.002** | **[-3.4e-03, -0.0001]** | **-2.12** | **0.037** |
| Random effects |  |  |  |  |
| Groups | Variance | Standard deviation |  |  |
| Participant (intercept) | 0.0005 | 0.023 |  |  |
| Participant (time) | 0.002 | 0.045 |  |  |
| Participant (intercept) | 0.0002 | 0.015 |  |  |
| Participant (condition) | 0.0009 | 0.030 |  |  |
| Residual | 0.0009 | 0.030 |  |  |
| Observations | 144 |  |  |  |
| Participants | 36 |  |  |  |

Table S11. Linear mixed model estimates for the median cortisol, Tone and Condition on the RT for *valence interference*, Study 2.

| Fixed effects |  |  |  |  |
| --- | --- | --- | --- | --- |
|  | b | CI_bo [95%] | t | p |
| (intercept) | 3.3e-02 | [0.02, 0.05] | 4.33 | 4.58e-05 |
| Tone order [Tone 2] | -6.8e-02 | [-0.09, -0.05] | -7.41 | 2.91e-10 |
| Condition [POS] | -3.5e-02 | [-0.06, -0.01] | -2.87 | 0.006 |
| Cortisol | 2.1e-04 | [-0.001, 0.001] | 0.33 | 0.74 |
| Tone order [Tone 2] by Condition [POS] | 7.3e-02 | [0.05, 0.09] | 7.05 | 1.17e-09 |
| Tone order [Tone 2] by Cortisol | -5.5e-05 | [-0.001, 0.001] | -0.07 | 0.94 |
| Condition [POS] by Cortisol | -2.5e-05 | [-0.002, 0.002] | -0.03 | 0.98 |
| Tone order [Tone 2] by Condition [POS] by Cortisol | -6.6e-04 | [-0.002, 0.0009] | -0.79 | 0.43 |
| Random effects |  |  |  |  |
| Groups | Variance | Standard deviation |  |  |
| Participant (intercept) | 0.0003 | 0.016 |  |  |
| Participant (time) | 0.001 | 0.033 |  |  |
| Participant (intercept) | 0.0008 | 0.029 |  |  |
| Participant (condition) | 0.003 | 0.058 |  |  |
| Residual | 0.001 | 0.031 |  |  |
| Observations | 144 |  |  |  |
| Participants | 36 |  |  |  |

Table S12. Linear mixed model estimates for the median alpha-amylase, Tone and Condition on the RT for *salience interference*, Study 2.

| Fixed effects |  |  |  |  |
| --- | --- | --- | --- | --- |
|  | b | CI_bo [95%] | t | p |
| (intercept) | 1.13e-02 | [-1.8e-03, 2.4e-02] | 1.68 | 0.096 |
| Tone order [Tone 2] | -2.43e-02 | [-4.4e-02, -2.8e-03] | -2.39 | 0.020 |
| Condition [LS] | -3.15e-02 | [-5.1e-02, -1.5e-02] | -3.56 | 0.000 |
| Alpha-amylase | 7.07e-05 | [-8.6e-05, 2.1e-04] | 0.96 | 0.34 |
| Tone order [Tone 2] by Condition [LS] | 6.64e-02 | [4.6e-02, 9.0e-02] | 6.34 | 2.11e-08 |
| Tone order [Tone 2] by Alpha-amylase | -1.89e-04 | [-4.1e-04, 3.8e-05] | -1.71 | 0.093 |
| Condition [LS] by Alpha-amylase | 3.21e-05 | [-1.5e-04, 2.3e-04] | 0.33 | 0.74 |
| Tone order [Tone 2] by Condition [LS] by Alpha-amylase | 3.17e-05 | [-1.8e-04, 2.6e-04] | 0.28 | 0.78 |
| Random effects |  |  |  |  |
| Groups | Variance | Standard deviation |  |  |
| Participant (intercept) | 0.0004411 | 0.02100 |  |  |
| Participant (time) | 0.0017644 | 0.04201 |  |  |
| Participant (intercept) | 0.0002092 | 0.01446 |  |  |
| Participant (condition) | 0.0008369 | 0.02893 |  |  |
| Residual | 0.0009849 | 0.03138 |  |  |
| Observations | 144 |  |  |  |
| Participants | 36 |  |  |  |

Table S13. Linear mixed model estimates for the median alpha-amylase, Tone and Condition on the RT for *valence interference*, Study 2.

| Fixed effects |  |  |  |  |
| --- | --- | --- | --- | --- |
|  | b | CI_bo [95%] | t | p |
| (intercept) | 3.30e-02 | [0.02, 0.05] | 4.33 | 4.66e-05 |
| Tone order [Tone 2] | -6.78e-02 | [-0.08, -0.05] | -7.38 | 3.14e-10 |
| Condition [LS] | -3.49e-02 | [-0.06, -0.009] | -2.86 | 0.006 |
| Alpha-amylase | 2.58e-06 | [-0.0002, 0.0001] | 0.03 | 0.97 |
| Tone order [Tone 2] by Condition [LS] | 7.34e-02 | [0.05, 0.09] | 7.02 | 1.32e-09 |
| Tone order [Tone 2] by Alpha-amylase | -5.58e-05 | [-0.0002, 0.0001] | -0.56 | 0.58 |
| Condition [LS] by Alpha-amylase | 3.66e-05 | [-0.0002, 0.0003] | 0.27 | 0.78 |
| Tone order [Tone 2] by Condition [LS] by Alpha-amylase | 2.83e-05 | [-0.0002, 0.0003] | 0.25 | 0.80 |
| Random effects |  |  |  |  |
| Groups | Variance | Standard deviation |  |  |
| Participant (intercept) | 0.0003 | 0.016 |  |  |
| Participant (time) | 0.001 | 0.032 |  |  |
| Participant (intercept) | 0.0008 | 0.029 |  |  |
| Participant (condition) | 0.003 | 0.058 |  |  |
| Residual | 0.001 | 0.031 |  |  |
| Observations | 144 |  |  |  |
| Participants | 36 |  |  |  |


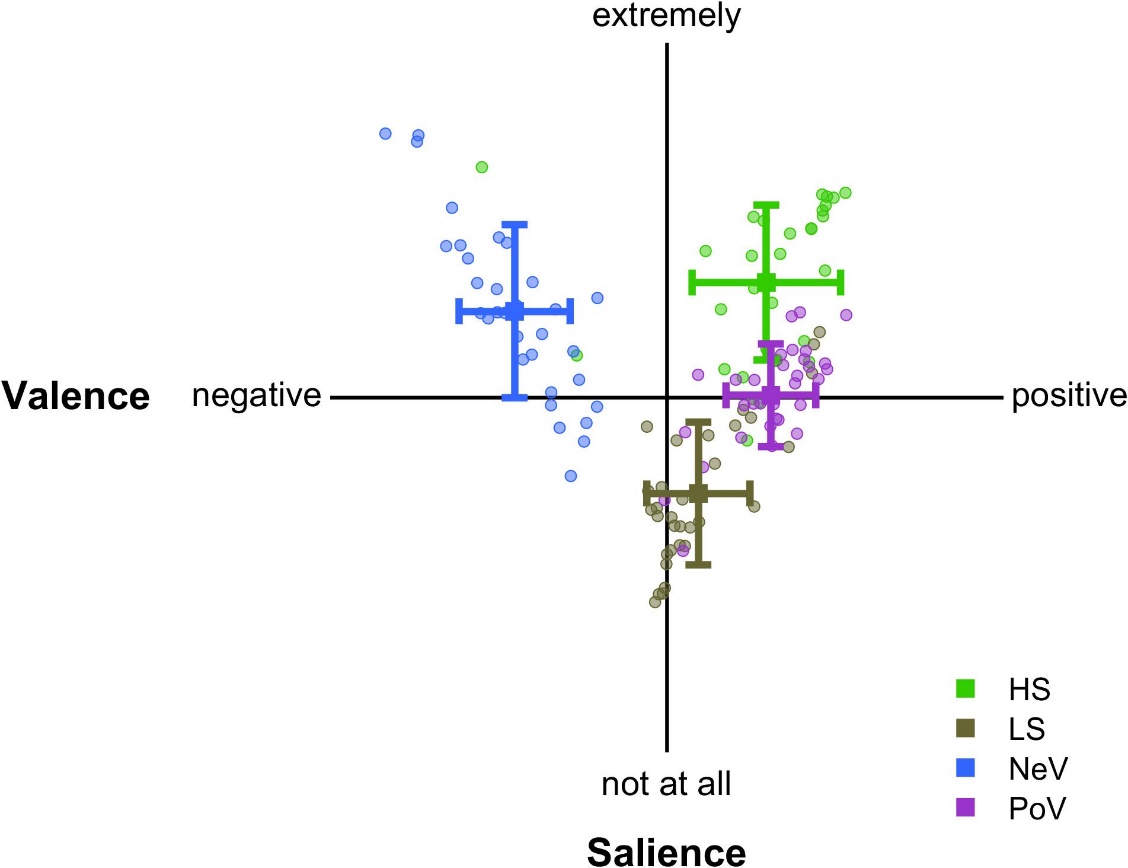


**Figure S1**. Mean ratings of each picture across the salience and valence dimensions. For *salience interference*, pictures were in the high salience (sea green) or low salience condition (old banana brown), while in the *valence interference* pictures were in the negative valence (parisian blue) or positive valence condition (orchid purple).
